# Editorial Note: Field and laboratory assessment of larvicidal activity of tobacco plants and the cigarette butt waste on *Culex pipiens* (Linnaeus, 1758), *Aedes aegypti* (Linnaeus, 1762) L. and non-target organisms

**DOI:** 10.1371/journal.pone.0353231

**Published:** 2026-07-07

**Authors:** The PLOS One Editors

The *PLOS One* Editors issue this Editorial Note to inform readers of concerns regarding compliance with PLOS Authorship policy for this article [1]. We regret that the issues were not addressed prior to the article’s publication.
